# Viscoelastic characterization of the lipid cubic phase provides insights into high-viscosity extrusion injection for XFEL experiments

**DOI:** 10.1038/s41598-025-25449-8

**Published:** 2025-11-07

**Authors:** Dmitrii Zabelskii, Ekaterina Round, Huijong Han, David von Stetten, Romain Letrun, Chan Kim, Tokushi Sato, Diogo V. M. Melo, Raphaël de Wijn, Konstantin Kharitonov, Peter Smyth, Katerina Doerner, Marco Kloos, Thomas Dietze, Luis Lopez Morillo, Richard Bean, Adam Round

**Affiliations:** 1 European XFEL GmbH, Schenefeld, Germany; 2 European Molecular Biology Laboratory, Hamburg Unit C/O DESY, Hamburg, Germany

**Keywords:** X-ray crystallography, Rheology

## Abstract

Serial crystallography (SX) is a powerful method for determining macromolecular structures, enabled by the advent of X-ray free-electron lasers (XFELs). SX experiments require the continuous delivery of microcrystals, achievable through liquid jets, high-viscosity extrusion (HVE) jets, and fixed-target methods. The lipid cubic phase (LCP) is a lipid-based medium commonly used for membrane protein crystallization and as a carrier medium for HVE injection. In this study, we present a rheometric characterization of LCP media and demonstrate that sample viscosity correlates well with the injection stability and, therefore, can effectively predict sample stability during HVE jetting. Using this approach, we determined the viscosity range corresponding to the stable, metastable, and unstable jetting regions. The critical viscosity values for the metastable and unstable jetting regions are 7 × 10^5^ and 10^4^ mPa⋅s, measured at the 0.3 s^-1^ shear rate. We show that ambient humidity is crucial for rheometric and fixed-target experiments involving LCP-embedded crystals. Specifically, LCP-embedded crystals rapidly lose diffraction quality when exposed to ambient humidity below 80%. Additionally, we demonstrate that sample viscosity measured in the rheometric experiment can help determine the necessary amount of stabilizing additive for HVE jet optimization. This approach was successfully tested on LCP mixed with long-chain polyethylene glycol and stabilized with Pluronic F-127 polymer.

## Introduction

X-ray free-electron lasers (XFELs) generate short, coherent X-ray pulses with peak brilliance surpassing third-generation synchrotron sources^1,2^. The application of XFEL radiation in macromolecular crystallography offers unique advantages over conventional synchrotron radiation. Firstly, XFEL pulses are typically generated with a temporal width of tens of femtoseconds, enabling time-resolved studies at the ultrafast timescale when coupled with femtosecond optical lasers. Secondly, intense XFEL pulses immediately destroy illuminated crystals, simultaneously providing the best available approximation to a radiation-damage-free structure due to the principle known as diffraction before destruction^3–7^. Consequently, a serial data-collection method is essential, with each X-ray exposure producing diffraction at a random orientation. Complete coverage of reciprocal space is achieved by collecting numerous diffraction patterns from thousands of microcrystals and merging many partial intensities. Serial crystallography (SX) relies on efficient sample replenishment^8,9^. One approach to SX experiments at XFELs is to use gas dynamic virtual nozzle (GDVN) injection to deliver crystals in a thin liquid jet surrounded by helium gas to prevent freezing if the experiment is conducted in a vacuum^10,11^. However, GDVN injectors consume large amounts of sample because the liquid microjet flows continuously at high speeds, while the pulsed FEL allows only sparse sampling. This inefficiency is significantly reduced by injecting crystals into a viscous material. High-viscosity extrusion (HVE) injectors operate at low flow rates, allowing most of the sample to be probed by XFEL pulses^12,13^. These injectors require embedding crystals in a viscous medium that provides stable jetting conditions. The continuous search for carrier media for microcrystals has yielded multiple suitable options, such as grease^14^, Vaseline^15^, polymer systems^16^, and other systems^17^. Another carrier medium widely used for HVE injection is lipid cubic phase (LCP)^18^.

The LCP is a highly ordered, three-dimensional structure formed by specific lipids, such as monoolein (MO, 1-Oleoyl-rac-glycerol), in the presence of water^19–22^. Such mesophases are characterized by their ability to self-assemble into a continuous, periodic structure, creating a network of interconnected water channels surrounded by lipid bilayers. The MO: water mixture can exist in different forms, forming lamellar (L_α_ and L_c_), bicontinuous cubic-Pn3m and Ia3d phases, hexagonal (H_2_), and fluid isotropic (L_2_) phases, depending on the temperature and water content^23^. The bicontinuous cubic phases are particularly useful for membrane protein crystallization (*in meso* crystallization), as they provide a membrane-like environment that supports these proteins’ proper folding and function. The high viscosity of LCP makes it a perfect carrier for membrane protein crystals grown *in meso*, allowing reliable injection under various conditions^24^. However, using LCP for crystals not grown *in meso* is limited due to compatibility and stability issues with commonly used crystallization additives, such as 2-methyl-2,4-pentanediol (MPD), reducing LCP-based media’s viscosity^25^.

Furthermore, for membrane protein crystals grown using surfactants, the detergent concentration around the protein molecules can decrease as the detergent partitions into the LCP, potentially destabilizing the protein and crystals. While the structural, morphological, optical, and thermal properties of LCP have been studied with great detail^22^, there are only a few studies on the viscoelastic properties of monoolein-based LCP systems^26^. The viscoelastic characteristics are particularly important for understanding jetting stability under different flow rates and ambient conditions. This study aims to comprehensively analyze the impact of common crystallization additives on the rheometric properties of monoolein-based media and their resulting jetting stability.

## Results

### Phase composition of the monoolein/water systems

Firstly, we tested the LCP without additives, which we denote as ‘pure’ LCP, composed of a 70:30 (v/v) MO: water mixture, which formed a transparent and birefringent medium corresponding to the Pn3m phase, consistent with the previous studies^24^. In this work, we use the (v/v) fraction rate instead of the (w/w) rate for LCP analysis since the MO: water mixing is typically done using coupled syringes, which allows easy estimation of the (v/v) fraction and not the (w/w) fraction. The density of monoolein at 35 °C is around 0.94 g/cm^3^, which allows for recalculation between concentration rates^27^. The 70:30 (v/v) MO: water composition is equivalent to the 68:32 (w/w) ratio, a typical ratio used for HVE injection, as a slight excess of lipid is preferred due to easier mixing and better homogenization of the LCP. The lipid cubic phase at room temperature (20 °C) is highly susceptible to ambient humidity and can dehydrate within minutes at less than 50% humidity levels. To maintain the water content of LCP, the measurements were taken at a set humidity level of 75%, both preventing samples from dehydration and absorbing excess water from the environment. Without humidity control, the LCP medium undergoes rapid dehydration, significantly altering the sample viscosity and leading to unreliable jetting. To ensure the repeatability of results, we performed a thixotropic test before all viscosity measurements.

**Fig. 1:**
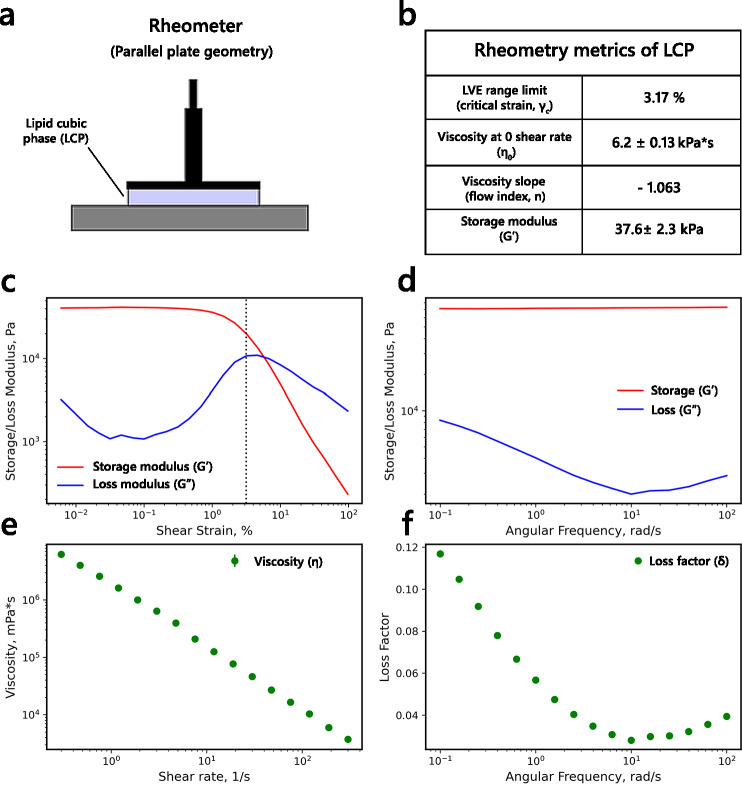
Rheometric characteristics of monoolein-based lipid cubic phase. (**a**). Rheometric setup used for the experiment. The Anton Paar MCR 92 rheometer was used for all rheometric experiments. (**b**). Summary of LCP (MO: water 70:30 (v/v)) rheometric features retrieved from the oscillation and rotational experiments. (**c**). The dependence of Storage (G′) and Loss (G′′) moduli on the shear strain (γ) at a constant angular frequency (ω, 10 Hz). (**d**). The dependence of Storage and Loss moduli on angular frequency at constant shear strain (0.1%). (**e**). Steady-state flow curve for LCP measured via rotational experiment. (**f**). The dependence of the Loss Factor (tan δ, G′/G′′) on the angular frequency. The n = 7 individual measurements were done.

All LCP-based systems followed the shear-thinning fluid flow behavior, characterized by decreased viscosity with increased applied shear stress. The ‘pure’ LCP followed the power-law decrease in viscosity (η = η_0·_γ^n^) with η_0_ = 6.2 ± 0.13 kPa·s and power-law flow index n = -1.06 (Fig. 1). The power-law index near the value of -1 indicates the ‘plastic’ behavior for the monoolein-based LCP, similar to other bicontinuous cubic phases^28^. This result also agrees well with the previous evaluation of the LCP viscosity, conducted at a slightly higher temperature (26 °C)^29^. The frequency-dependent behavior of the LCP was measured using amplitude (AS) and frequency sweep (FS) experiments. In the AS experiment, the ‘pure’ LCP followed a linear viscoelastic behavior (LVE) up to 3% critical shear strain (LVE range limit). Afterward, the Storage modulus (G’) sharply declined, indicating a stress-induced loss of energy stored in the system and, therefore, a loss of internal structure. The Loss modulus (G’’) behavior is biphasic, with the initial increase indicating the increase in energy dissipation upon shear stress increase. After reaching the critical shear strain, the G’’ decreases, which reflects a breakdown of the material’s structure, where the ability to dissipate energy via viscous mechanisms diminishes. Therefore, beyond the LVE region, the LCP likely undergoes a partial transition to the liquid crystalline phase (Lc), consistent with the previous observations of LCP phase behavior during HVE injection both in vacuum and air^30^. Interestingly, the cubic phase can recover after the applied stress, returning to the initial viscosity and phase state. In the FS experiment at shear strain (0.1%) within the LVE range, the G′ modulus remains constant. Thus, the deformation energy caused by shear stress remains in the system, and therefore, the LCP shows a completely elastic behavior under low shear stress conditions. The ability of the LCP to preserve its internal organization under low and mild stress conditions facilitates the continuity of the flow, a key feature for practical applications. Importantly, the LCP can embed protein crystals, providing an efficient buffer against shear stress.

### Influence of lipid content on LCP viscosity

**Fig. 2:**
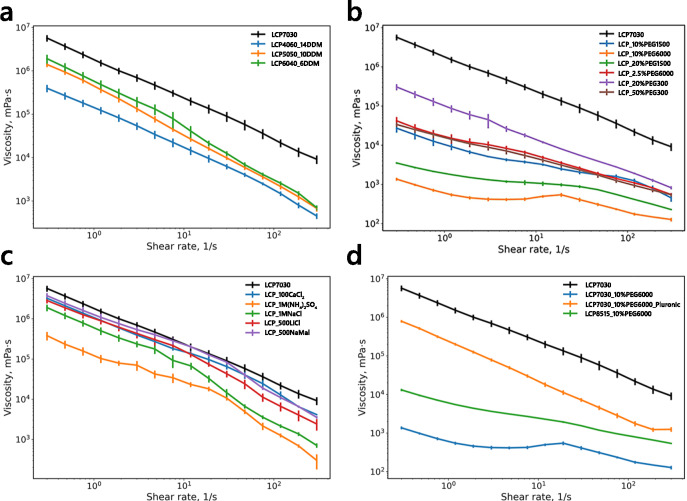
Influence of different additives on LCP viscosity. (**a**). Influence of the water content on the viscosity of the lipid cubic phase. Viscosity curves of LCP media supplemented with (**b**). polyethylene glycol (PEGs) and (**c**). salt additives. (**d**). Viscosity curves of the LCP that was softened with PEG 6000 and then supplemented with different stabilizing agents. The viscosity curve corresponding to the pure LCP (MO: water 70:30 (v/v)) was added to all subfigures as a reference. The additive percentage was calculated relative to the water fraction of the LCP.

Next, we evaluated how a decrease in the lipid content changes the lipid phase viscosity. While the phase behavior of MO: water mixtures has been thoroughly studied^22^, the viscosity profiles of the different phases were not reported. Under room temperature conditions, the MO: water mixture can employ four different phase states: the lamellar phases (La and Lc, > 80% MO (w/w)) at high lipid content, cubic gyroid (Ia3d, 65–75% MO (w/w)) phase, and cubic primitive (Pn3m, 40–42% (w/w)). The Pn3m phase is the most commonly used lipid phase for membrane protein crystallization due to its microstructure, allowing the formation of continuous water channels^25^. Adding more water to the Pn3m phase does not lead to phase rearrangement but rather to phase separation, where the Pn3m phase coexists with water. The phase diagram significantly shifts with the introduction of detergent in the system. Adding maltoside detergents, such as DDM (N-Dodecyl-β-D-maltoside), causes the concentration-dependent phase transition to first Ia3d and then the lamellar phase, allowing the lipid phase to absorb more water from the surroundings. To understand the rheological properties of LCP with reduced lipid content, we tested three samples, namely 60:40, 50:50, and 40:60 MO: water (v/v), supplemented with 6%, 10%, and 14% DDM (v/v), respectively, to avoid phase separation (Fig. 2a). The non-modified LCP (70:30 MO: water, v/v) was used as a reference. The decrease in lipid content caused phase softening, reducing η_0_ by at least one order of magnitude, which is consistent with the assumption about the partial phase transition.

### Influence of precipitants on LCP viscosity

To evaluate the impact of different common additives on lipid phases, we selected a set of salts (NaCl, CaCl_2_, LiCl, (NH_4_)_2_SO_4_) and organic compounds, such as polyethylene glycols (PEG), 2-Methyl-2,4-pentanediol (MPD), and sodium malonate (NaMal), commonly used for crystallization^31^. While all these additives are miscible with LCP at room temperature, the addition of some compounds, such as PEGs and MPD, causes concentration-dependent phase ‘swelling’, which can cause significant loss of order and ‘softening’ of the LCP^22^. While the dilation of the internal structure can benefit the crystallization of some membrane proteins, the reduction in viscosity can be detrimental to the HVE injection stability. We used saturating concentrations for each precipitant to evaluate a qualitative impact on LCP viscosity. The results are presented in Fig. 2c. The common salts, such as NaCl, LiCl, and CaCl_2_, as well as NaMal, had a modest softening effect on LCP viscosity, decreasing η_0_ by less than one order of magnitude. Interestingly, the addition of 1 M NaCl had a more substantial softening effect at the high shear rates (> 10 s^-1^), reducing the LCP viscosity by one order of magnitude in that region. The ammonium sulfate had a more pronounced effect, reducing the η_0_ by roughly one order of magnitude, the highest among the tested salts. Notably, all results are consistent with the small-angle scattering experiments (SAXS), which show that common salts can slightly change the lattice parameters of LCP, but do not induce phase transitions^25^.

**Fig. 3:**
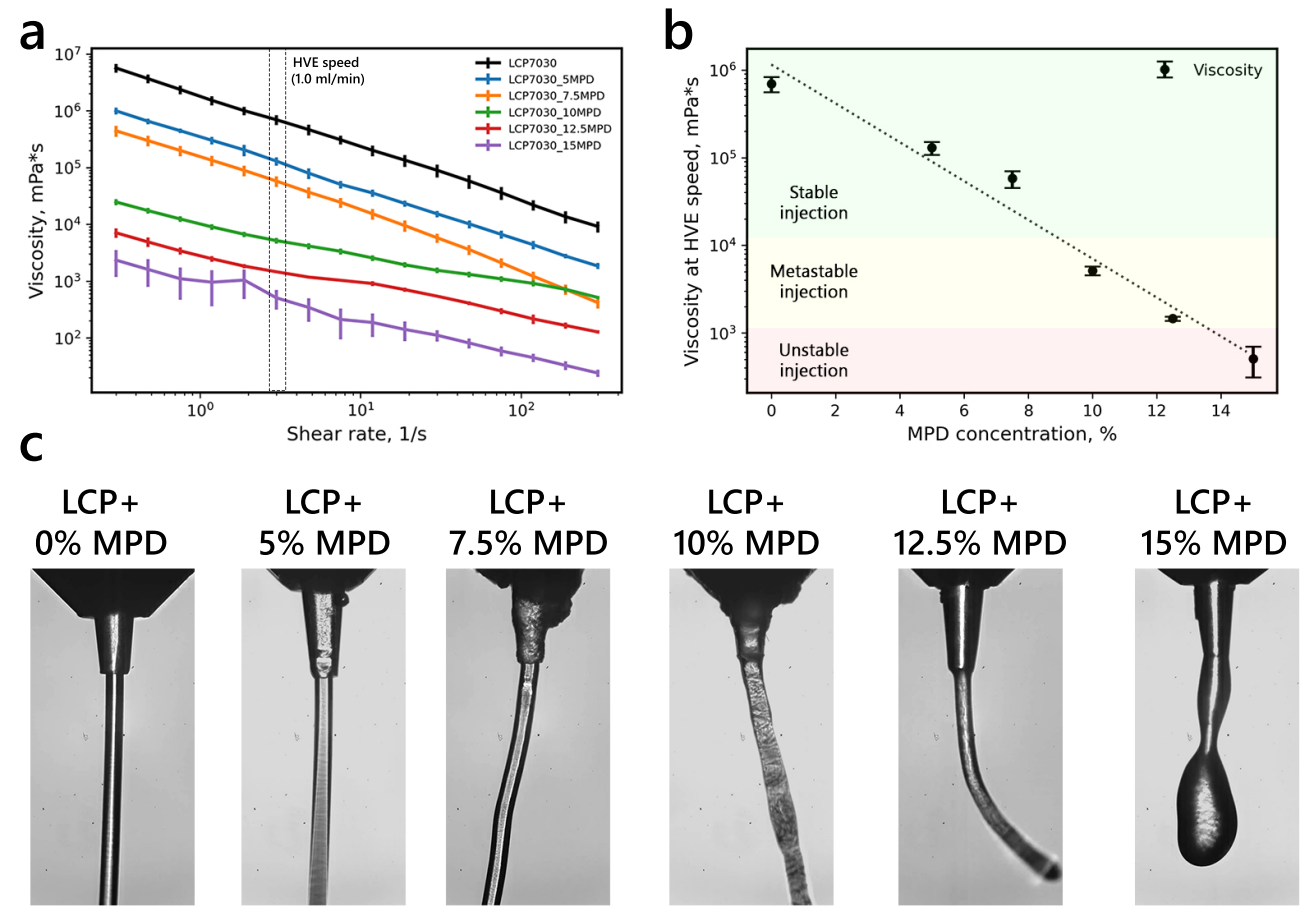
Impact of viscosity on the injection stability. (**a**). Viscosity curves of the LCP supplemented with different concentrations of MPD additive. (**b**). Dependence of the viscosity at HVE speed (shear rate of 47.5 s^-1^) on the MPD additive concentration. (**c**). Representative images of the HVE injection of LCP supplemented with different concentrations of MPD. All photos show the sample extruded from a nozzle with a 100 μm diameter at the 1.0 μL/min jetting speed. The additive percentage was calculated relative to the water fraction of the LCP.

The impact of MPD and PEGs on LCP viscosity was more pronounced. The addition of 10% of MPD (v/v) resulted in an almost two-order-of-magnitude decrease in the LCP viscosity (Fig. 3). Similarly, all tested PEGs have induced a significant reduction in the viscosity, which is, again, in agreement with the SAXS experiments, which indicate that PEGs and other organic compounds, such as butanediol and hexanediol, cause ‘swelling’ and subsequently, LCP phase transition from the cubic Pn3m phase to the lamellar phase (L_α_ and L_c_)^22,25,32^. PEGs and many other organic compounds can bind water molecules, reduce their activity, and dramatically influence lipid-based phases. We tested whether the PEGs’ influence on the LCP phase depends on the polymer’s molecular weight (MW) or if the effect is purely concentration-driven. We measured LCP, supplemented with the PEGs of different MW (300, 1500, and 6000), that were concentration-adjusted (Fig. 2b). Interestingly, the three additives: 50% PEG 300 (v/v), 10% PEG 1500 (v/v), and 2.5% PEG 6000 (v/v), all resulted in nearly similar viscosity profiles, which indicates that the LCP viscosity reduction caused by PEGs is proportional to the number of water molecules bound by the PEG’s ethylene oxide group, or, more practically, a product of the PEG concentration and MW. Therefore, while all PEGs are capable of LCP ‘swelling’, the long-chain PEGs are specifically problematic due to their high MW values in terms of HVE jetting stabilization.

### Rheological limits of HVE injection

The HVE injection method is beneficial for sample-efficient membrane protein studies and particularly useful for serial crystallography applications. The initial standard prerequisite before any SX experiment is successful injection testing (for both high- and low-viscosity methods). Determination of whether crystals of the sample of interest embedded in the carrier matrix can be reliably used for injection often requires a significant amount of trial and error. Jet stability plays a critical role in the successful SX experiment, as an unstable jet can significantly alter jet speed and the overlap between the jet and X-ray beam, affecting the hit rate and the time delay in pump-probe experiments. Injection testing includes jet speed and diameter evaluation, often done by analyzing videos of the jet and images recorded with high-speed cameras^11^. In this work, we propose that sample viscosity, measured in a rheometry experiment, can also serve as a helpful metric for evaluating jet stability. We measured HVE injection and viscosity curves for LCP samples supplemented with different concentrations of another common organic precipitant, 2-Methyl-2,4-pentanediol (MPD), to determine the correlation between measured viscosity and jet stability. MPD is a compound with surfactant properties and a common chemical additive in different crystallization techniques. While MPD is also used for *in meso* crystallization, it significantly reduces the viscosity of the LCP in a concentration-dependent manner and was shown to ‘spongify’ the lipid mesophase, changing its phase diagram^22^. We selected MPD for the test as it allows probing a wide viscosity range with relatively low additive concentrations^33^. We tested six samples of LCP with added MPD in the 0–15% (v/v) range with 2.5% (v/v) steps. The results are presented in Fig. 3. The measured viscosity curves show a concentration-dependent reduction of LCP viscosity consistent with the visual inspection of LCP behavior upon sample loading. In parallel, the same sample was tested at a jet testing station using the 100 μm nozzles (see full details in Methods). All samples were measured with a similar setup under three different flow rates: 0.5, 1.0, and 1.5 μL/min, focused by a helium stream of 10 mg/min. Consistent with the rheological testing, the unmodified LCP showed stable jetting and was unaffected in jet pointing and speed. While the LCP supplemented with 5% MPD (v/v) was also injected well, the 7.5% MPD (v/v) sample was jetted with variable jet pointing. LCP samples supplemented with higher concentrations of MPD showed unstable pointing and jet speed, and the 15% MPD (v/v) sample did not form a jet, producing droplets of variable size. We classified the observed injection into three categories: stable injection if the jet had both stable pointing and speed; metastable injection if the jet had instabilities that could be mitigated with helium focusing and/or jet speed increases; and unstable injection if the jet was impossible to stabilize with helium focusing. During stable HVE injection, the lamellar flow of the medium is observed, and the average shear rate inside the stream can be calculated using the Hagen/Poiseuille relation ${\gamma }_{a}=(4\cdot Q)/(2\pi \cdot {R}^{3})$, where Q is the volumetric flow rate. With the 100 μm nozzle and the stable flow rate of 0.5 μL/min, the average shear rate was around 10^2^ s^-1^. To understand the relationship between viscosity and jet stability, we selected two shear rate regions: an LVE shear region of 0.3 s^-1^ and a jet shear region of 75 s^-1^. Interestingly, the viscosity values measured at both LVE and jet shear rates show a linear dependence on MPD concentration, which allows for the interpolation analysis of jet stability. Although the HVE can reach higher shear rates (> 100 s^-1^), where the LCP undergoes a partial phase transition, it can be more beneficial to estimate the sample viscosity at lower shear rates, where the LCP is more stable and therefore, the rheometric characterization would be more accurate. Nevertheless, it is possible to determine the border values of viscosity for injection stability at both 0.3 and 75 s^-1^, as well as at other shear rate values. At the 0.3 s^-1^ shear rate, the medium with a viscosity lower than 7 × 10^5^ mPa⋅s will likely produce metastable injection. At a viscosity lower than 10^4^ mPa⋅s, the sample jet becomes unstable. At the 75 s^-1^ shear rate, the metastable and unstable jet regimes correspond to viscosities of 2 × 10^4^ and 3 × 10^3^ mPa⋅s, respectively (Fig. 3b).

### Stabilization of the softened LCP media with additives

The crystallization conditions used for *in meso* crystallization largely depend on the nature of the protein, which can lead to varied viscosity of the LCP media where the crystals appear. Many common precipitants and detergents discussed in previous sections can significantly lower the viscosity of the LCP. In some cases, the lower viscosity of the carrier medium can be mitigated by jetting at an increased flow rate and/or focusing the jet with a sheath gas. However, a more reliable way of jet stabilization is supplementing the carrier media with a stabilizing agent, a viscous additive, that can increase the viscosity of the jet at the cost of diluting the sample. The most common additive used for such a purpose is monoolein itself, which is chemically neutral to the sample, as it already embeds the crystals. Recently, some alternative agents have been introduced for a similar purpose, namely, sodium carboxymethylcellulose (NaCMC), grease, agarose, and polymers such as Pluronic F-127^34^. Despite their utility for jet stabilization, the commonly used chemicals vary in their chemical compatibility, X-ray background, and compatibility with the experimental setup. This study focused on characterizing two common stabilizing agents, monoolein and thermoreversible hydrogel Pluronic F-127, commonly used at the SPB/SFX instrument of European XFEL^2,28^. We supplemented a highly softened LCP media (LCP mixed with 10% PEG 6000 (v/v)) that was further mixed with a stabilizing agent in a 1:1 ratio. The added monoolein was pre-heated to 42 °C before mixing with the LCP media. The 35% Pluronic: water mixture (w/w) was prepared as previously described. Both stabilizing agents were thoroughly mixed with the softened LCP in coupled Hamilton syringes before the rheometric experiments. The viscosity curve experiments were done for viscosity evaluation (Fig. 2d). The softened LCP had a viscosity of (5.2 ± 0.7) × 10^2^ mPa⋅s at HVE injection speed, corresponding to an unstable injection. The additions of both MO and Pluronic had a significant effect on the viscosity of the softened LCP, however, to a different degree. The viscosity of the LCP supplemented with MO increased by a factor of ten to (7.2 ± 0.7) × 10^3^ mPa⋅s, corresponding to metastable injection conditions. The addition of Pluronic F-127 had a much more pronounced stabilization effect, increasing the viscosity of LCP to (2.2 ± 0.7) × 10^5^ mPa⋅s, corresponding to stable injection.

### Impact of dehydration and additives on diffraction data

**Fig. 4:**
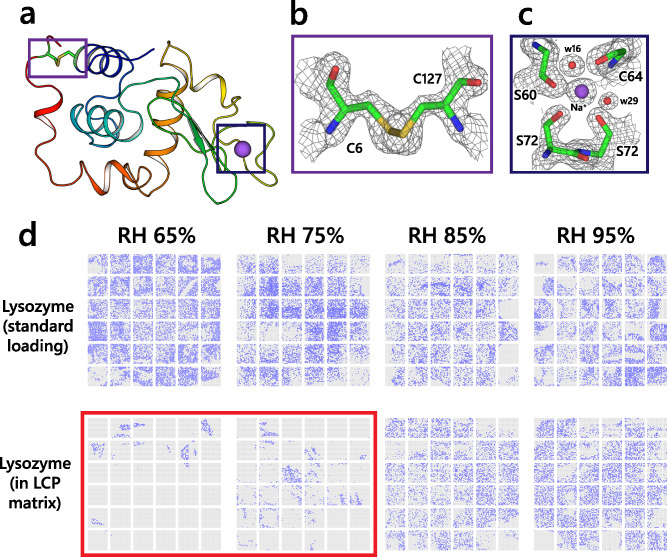
Lysozyme structures at different RH conditions. (**a**). Cartoon representation of the hen egg white lysozyme structure. (**b**). 2F_c_-F_o_ electron density maps corresponding to the C6–C127 disulfide bond. The residues are drawn as sticks. (**c**). 2F_c_-F_o_ electron density maps corresponding to the sodium binding site in the lysozyme structure. The maps are contoured at a 1.5σ level. (**d**). The hit maps of the individual chips used for data analysis are shown. The indexed hits are marked with the blue dots. The chips measured for LCP-embedded lysozyme at 65% and 75% RH are highlighted in red. The highlighted chips were not processed due to the lack of hits.

**Fig. 5:**
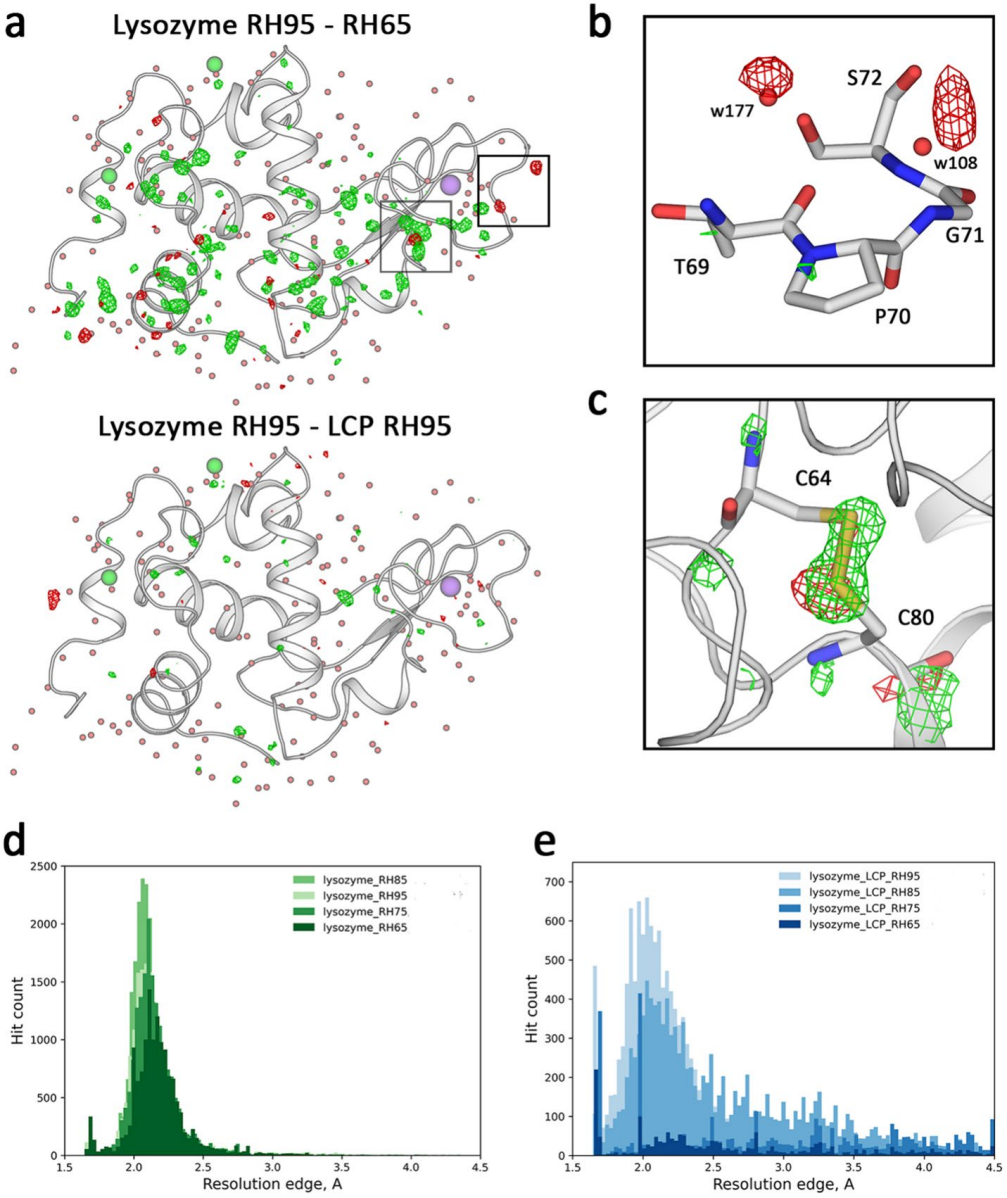
Differences between lysozyme datasets. (**a**). Fo-Fo difference maps overlaid on lysozyme structure (RH95) between the RH95 and RH65 conditions (top) and between the RH95 conditions in mother liquor and embedded in LCP medium. The maps are contoured at the 3.5σ level. (**b**). An example of water displacement is associated with the change in humidity. The difference map is contoured at the 3σ level. (**c**). An example of a side chain rearrangement upon humidity change. The difference map is contoured at the 3σ level. (**d**). Distribution of the resolution limit of the individual crystals is shown at different humidity levels in the mother liquor. (**e**). The distribution of the resolution limit of the individual crystals embedded in an LCP medium.

**Fig. 6:**
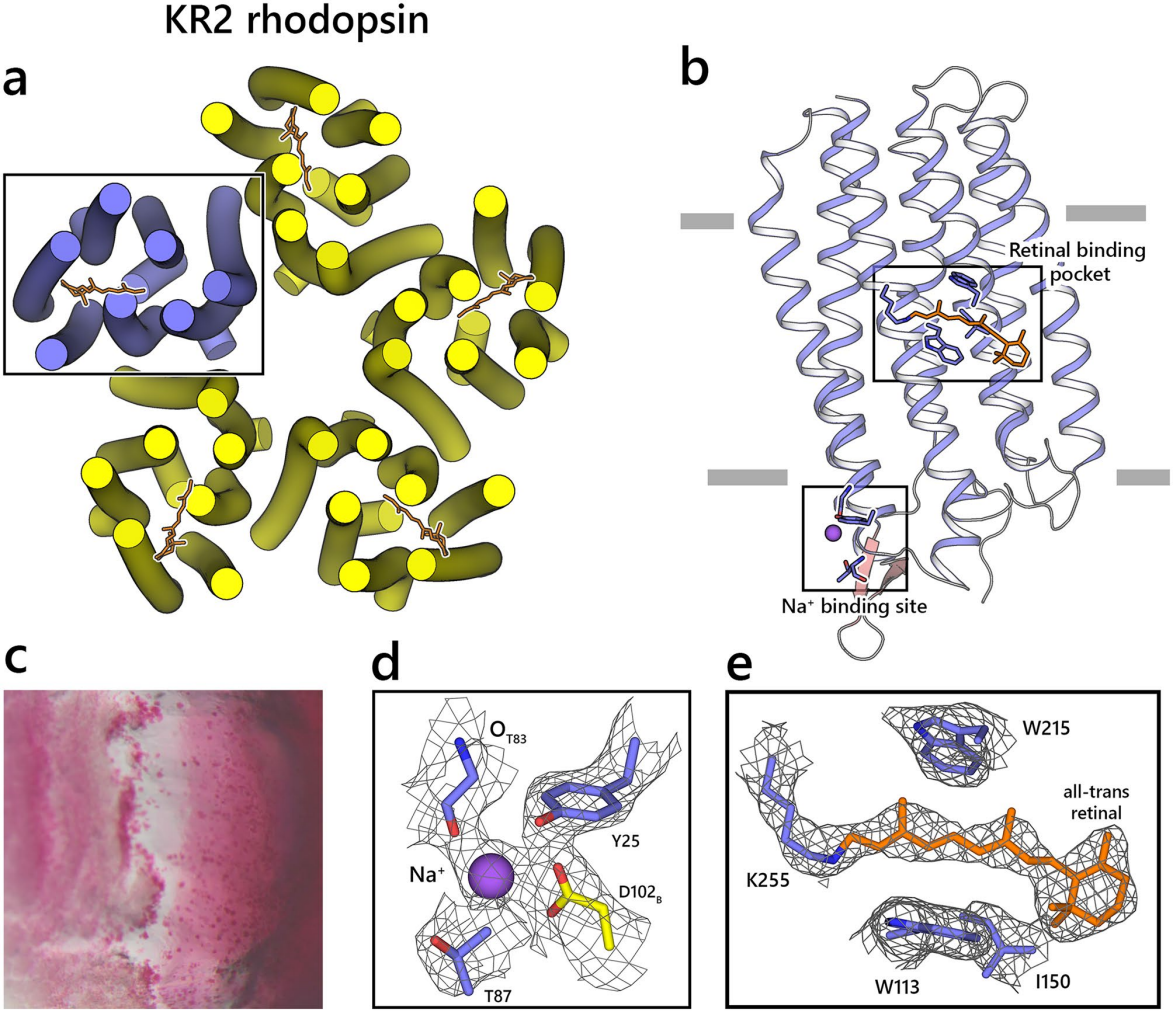
Room temperature structure of KR2 rhodopsin. (**a**). Overall view on the KR2 rhodopsin pentamer structure (RH 85%). Protein helices are shown as cylinders, and retinal cofactors are shown as sticks. (**b**). Close-up view of the KR2 monomer structure viewed parallel to a membrane. Membrane boundaries are shown. (**c**). Examples of the KR2 crystals grown at room temperature in PCR tubes. d. 2F_c_-F_o_ electron density maps corresponding to the sodium binding site. e. 2F_c_-F_o_ electron density maps corresponding to the retinal cofactor. The maps are contoured at 1.2σ level.

**Fig. 7:**
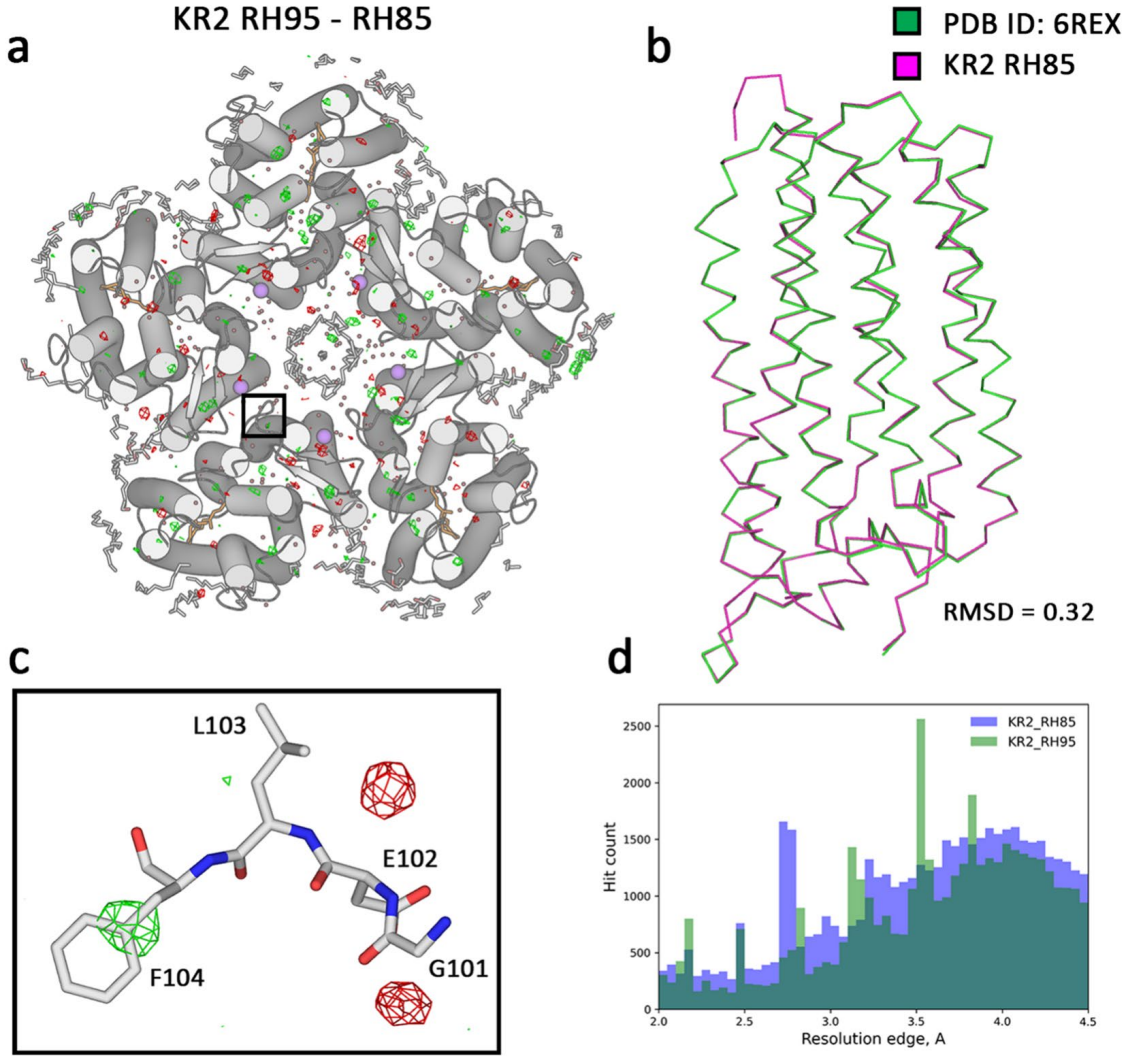
Differences between KR2 datasets. (**a**). Fo–Fo difference maps overlaid on KR2 structure (RH85) between the RH95 and RH85 conditions. The maps are contoured at 3.5σ level. (**b**). Ribbon comparison between the main chain Ca atom positions between KR2 RH85 structure and cryo-structure of KR2 (PDB ID: 6REX). (**c**). A close-up view of a side-chain rearrangement of KR2. The difference map is contoured at 3σ level. d. The distribution of the resolution limit of the individual crystals is shown at different humidity levels.

We studied the influence of different humidity environments on the quality of crystallographic data. For that, we used two samples: hen egg white lysozyme crystals, commonly used as a crystallographic standard^35–38^, and a membrane protein, pentameric light-gated sodium pump KR2 from *Krokinobacter eikastus*^39–44^. Both samples were measured using the fixed target setup at the P14.2 endstation of the PETRAIII synchrotron in Hamburg, Germany. The lysozyme crystals were measured in their native conditions and embedded in an LCP medium. For the experiment, four different relative humidity (RH) settings were used, namely 65%, 75%, 85%, and 95%. For the KR2 sample, we collected the datasets for 85% and 95% RH conditions. We could not collect diffraction data under lower humidity due to sample dehydration, which detrimentally affected diffraction quality. The results are presented at Figs. 4–7. Full details of the sample preparation and measurements can be found in Methods.

Under all conditions, the lysozyme crystals diffracted beyond the detector edge (Table 1). We compare the dataset at the detector edge cut-off, 1.7 Å, which is limited by the sample chamber geometry. When loaded on a chip in its crystallization buffer, the crystals remained stable under all RH conditions. They did not exhibit signs of dehydration, which is consistent with previously reported data^45^. We also did not observe any change in the cell parameters of the lysozyme upon changing the relative humidity. The structure of lysozyme was similar under all RH conditions and is close to previously reported structures for lysozyme at room temperature^36^. The only noticeable difference between the structures was the number of observed water molecules, positively correlated with relative humidity (Table 2). Under all RH conditions, the crystals show a nearly similar distribution of the maximum resolution, with a sharp peak at the detector cut-off value (Fig. 5).

**Table 1:** Rheometric viscosity of LCP with different additives.

	Additive percentage*	Viscosity at 0.3 s^-1^ shear rate (mPa·s)	Viscosity at 75.4 s^-1^ shear rate (mPa·s)		Injection stability
LCP no additive**	N/A	(5.7 ± 0.9)·10^6^	(3.6 ± 0.09)·10^4^	+	
LCP 20% PEG 300	6%	(1.8 ± 0.7)·10^5^	(2.9 ± 0.04)·10^3^	+	
LCP 50% PEG 300	15%	(3.7 ± 0.4)·10^4^	(1.3 ± 0.2)·10^3^	-	
LCP 10% PEG 1500	3%	(2.7 ± 0.6)·10^4^	(1.6 ± 0.2)·10^3^	-	
LCP 20% PEG 1500	6%	(3.4 ± 0.2)·10^3^	(5.2 ± 0.03)·10^2^	-	
LCP 2.5% PEG 6000	0.75%	(3.1 ± 0.7)·10^4^	(1.5 ± 0.1)·10^3^	-	
LCP 10% PEG 6000	3%	(1.4 ± 0.1)·10^3^	(2.3 ± 0.2)·10^2^	–	
LCP 5% MPD	1.5%	(9.8 ± 1.1)·10^5^	(6.8 ± 0.7)·10^3^	+	
LCP 7.5% MPD	2.25%	(5.4 ± 0.7)·10^5^	(2.5 ± 0.3)·10^3^	+	
LCP 10% MPD	3%	(2.4 ± 0.4)·10^4^	(1.1 ± 0.2)·10^3^	-	
LCP 12.5% MPD	3.75%	(7.5 ± 1.2)·10^3^	(2.8 ± 0.2)·10^2^	-	
LCP 15% MPD	4.5%	(1.3 ± 0.7)·10^3^	(7.5 ± 1.2)·10^1^	–	
LCP 100 mM CaCl_2_	30 mM	(3.2 ± 0.5)·10^6^	(2.4 ± 0.5)·10^4^	+	
LCP 1 M (NH_4_)_2_SO_4_	300 mM	(3.8 ± 0.9)·10^5^	(2.1 ± 0.4)·10^3^	-	
LCP 1 M NaCl	300 mM	(1.9 ± 0.4)·10^6^	(3.6 ± 0.04)·10^4^	+	
LCP 500 mM LiCl	150 mM	(2.9 ± 0.4)·10^6^	(1.1 ± 0.02)·10^4^	+	
LCP 500 mM sodium malonate	150 mM	(3.8 ± 0.6)·10^6^	(1.8 ± 0.02)·10^4^	+	
LCP 60/40 (v/v) 6% DDM	2.4%	(1.9 ± 0.4)·10^6^	(4.1 ± 0.4)·10^3^	+	
LCP 50/50 (v/v) 10% DDM	5%	(1.4 ± 0.2)·10^6^	(3.6 ± 0.4)·10^3^	+	
LCP 40/60 (v/v) 14% DDM	8.4%	(3.9 ± 0.8)·10^5^	(2.5 ± 0.2)·10^3^	-	
LCP 85/15 (v/v) 10% PEG 6000	1.5%	(1.3 ± 0.09)·10^4^	(9.5 ± 0.2)·10^2^	-	
LCP 10% PEG 6000 + 50% Pluronic F-127	1.5%/17.5%***	(7.9 ± 0.6)·10^5^	(2.8 ± 0.4)·10^3^	+	

Injection stability is evaluated by comparison with the unmodified LCP. Each dataset is assigned a stable ( +), metastable (-), or unstable (–) jet regime. The additive percentage was calculated relative to the water fraction of the LCP. The total additive percentage calculated relative to the combined volume is presented.

* The additive percentage in % is the volume % (v/v), where applicable.

** The unmodified LCP (70/30 (v/v) MO: water) was used as a reference for injection stability evaluation.

*** The 35% Pluronic F-127 (w/w) was mixed with LCP in a 1:1 ratio (v/v).

**Table 2:** Data collection and refinement statistics for datasets from crystals not embedded in LCP.

	Lysozyme RH95	Lysozyme RH85	Lysozyme RH75	Lysozyme RH65
Data collection				
Indexed crystals	8901	4404	18,732	13,878
Space group	P4_3_2_1_2	P4_3_2_1_2	P4_3_2_1_2	P4_3_2_1_2
Cell dimensions				
*a*, *b*, *c* (Å)	79.0, 79.0, 38.0	79.0, 79.0, 38.0	79.0, 79.0, 38.0	79.0, 79.0, 38.0
α,β,γ (°)	90, 90, 90	90, 90, 90	90, 90, 90	90, 90, 90
Resolution (Å)	39.5 – 1.7 (1.76 – 1.7)	39.5 – 1.7 (1.76 – 1.7)	39.5 – 1.7 (1.76 – 1.7)	39.5 – 1.7 (1.76 – 1.7)
*R*_*split*_	16.35(64.17)	23.07(93.53)	10.4(34.917)	11.05(42.12)
*I* / σ*I*	5.22(1.58)	3.65(1.15)	8.35(2.77)	7.62(2.5)
Completeness (%)	100(100)	99.99(100)	99.99(100)	99.97(99.8)
Redundancy	124.9(73.5)	69.5(40.8)	301.3(178.2)	234.4(138.1)
Refinement				
Resolution (Å)	39.5 – 1.7	39.5 – 1.7	39.5 – 1.7	39.5 – 1.7
No. reflections	13,756 (1344)	13,755 (1344)	13,755 (1344)	13,753 (1341)
*R*_work_ / *R*_free_	15.96/19.87	16.1/19.55	15.77/19.69	16.12/19.87
No. atoms				
Macromolecules	1103	1113	1094	1080
Ligand/ion	18	18	22	22
Water	140	140	131	122
*B*-factors				
Macromolecules	22.19	22.97	21.10	20.55
Ligand/ion	43.30	44.68	37.80	37.29
Water	41.83	43.16	38.67	36.75
R.m.s. deviations				
Bond lengths (Å)	0.008	0.013	0.013	0.016
Bond angles (°)	0.98	1.45	1.31	1.52

*Values in parentheses are for highest-resolution shell.

In contrast, when the lysozyme crystals were embedded in the LCP matrix, the resolution distribution had a less defined peak (Fig. 5e). It also contained a significant fraction of crystals that diffracted worse than 2.5 Å, which indicates that LCP can negatively affect the diffraction quality of the crystals that were not grown using the *in meso* approach. Despite this, the crystallographic structures for lysozyme in native conditions and when embedded in LCP both have a comparable quality, which suggests that the adverse effects of LCP embedding can be considered minor or even negligible. Also, decreasing the relative humidity to below 80% caused a lipid phase transition that suppressed crystal diffraction. Interestingly, the LCP-embedded crystals diffracted even when loaded and measured under 65% and 75% humidity. However, the chip hit map shows that under both conditions, the crystals were clustered, indicating it is the phase transition and not the humidity that is detrimental to the crystal integrity (Fig. 4). Most likely, these crystals were localized in the areas where the LCP was not yet fully dehydrated, and therefore, the crystals were locally preserved. The structures of lysozyme embedded in LCP, both at 85% and 95% RH, are similar to the lysozyme structure at 95% RH without LCP. Also, the datasets of lysozyme with and without LCP show little difference in crystallographic quality metrics from the crystal, indicating that overall, LCP does not influence crystal diffraction quality (Tables 2 and 3). Examples of the ED maps can be found in Figs. 4 and 6.

**Table 3:** Data collection and refinement statistics for datasets from crystals embedded in LCP.

	Lysozyme LCP RH95	Lysozyme LCP RH85	Lysozyme HVE XFEL	KR2 RH95	KR2 RH85
Data collection					
Indexed crystals	7540	5723	20,460	7793	10,262
Space group	P4_3_2_1_2	P4_3_2_1_2	P4_3_2_1_2	C222_1_	C222_1_
Cell dimensions					
*a*, *b*, *c* (Å)	79.0, 79.0, 38.0	79.0, 79.0, 38.0	79.0, 79.0, 38.0	136.1, 240.9, 138.5	136.1, 240.9, 138.5
α,β,γ (°)	90, 90, 90	90, 90, 90	90, 90, 90	90, 90, 90	90, 90, 90
Resolution (Å)	39.5 – 1.7 (1.76 – 1.7)	39.5 – 1.7 (1.76 – 1.7)	19.23 – 1.7 (1.76 – 1.7)	68.05 – 3.0 (3.11 – 3.0)	68.05 – 3.0 (3.11 – 3.0)
*R*_*split*_	22.32(88.54)	24.76(91.37)	15.43(46.47)	36.46(130.75)	34.71(115.92)
*I* / σ*I*	4.02(1.07)	3.65(1.04)	6.87(2.95)	2.65(0.86)	2.88(0.95)
Completeness (%)	99.99(100)	99.99(100)	99.9(100)	99.92(99.7)	99.94(100)
Redundancy	71.2(42.1)	49.3(29.2)	267.3(195.8)	89.0(61.4)	101.3(69.6)
Refinement					
Resolution (Å)	39.5 – 1.7	39.5 – 1.7	22.5 – 1.7	68.05 – 3.0	68.05 – 3.0
No. reflections	13,756 (1344)	13,756 (1344)	13,770 (1347)	45,862 (4499)	45,868 (4512)
*R*_work_ / *R*_free_	16.86/19.97	16.7/19.54	16.7/19.9	17.26/20.61	17.44/19.77
No. atoms					
Macromolecules	1097	1097	1102	10,636	10,640
Ligand/ion	5	5	12	1099	1110
Water	164	152	151	204	182
*B*-factors					
Macromolecules	22.26	22.96	24.53	52.65	53.37
Ligand/ion	32.69	31.59	44.25	70.95	72.97
Water	42.54	42.95	41.16	56.78	53.56
R.m.s. deviations					
Bond lengths (Å)	0.004	0.004	0.016	0.003	0.003
Bond angles (°)	0.72	0.68	1.39	0.63	0.63

*Values in parentheses are for the highest-resolution shell.

The KR2 crystals were grown using *in meso* approach in the monoolein-based matrix, similar to the previously reported protocols^42,43^. The results are presented in Figs. 6 and 7. The retinal cofactors and sodium ions are present in the structure in similar conformations as in the 100 K structures, showing that structures at room temperature represent the functional state of KR2 (Fig. 6). The resolution distribution of the KR2 crystals showed a Gaussian-like distribution, with the best crystals diffracting up to 2.5 Å and the peak around 4 Å resolution. Both room temperature (RT) structures of KR2 were nearly similar to the KR2 structure measured from the single crystal at the 100 K temperature^42^. The crystal lattice of the KR2 crystals showed almost no change, with the root mean square deviation (RMSD) of 0.3 between RT and 100 K structures (Fig. 7). Also, the structures of the KR2 at 85% and 95% relative humidity were nearly identical, indicating that membrane protein crystals in LCP are stable within this hydration range.

## Discussion

### HVE jet stabilization tools for the ‘swollen’ LCP media

This work presents the viscoelastic characterization of the monoolein-based LCP medium, a common carrier medium in HVE injection widely used in serial crystallography experiments. This information complements the existing SAXS data on LCP phase composition with different additives^25^, and provides a valuable reference to the potentially problematic additives commonly used in crystallization buffers. In particular, we show that most inorganic salts widely used for crystallization do not significantly affect LCP viscosity, with the exceptions of NaCl and (NH_4_)_2_SO_4,_ which both reduce the viscosity by one order of magnitude in the jet shear region, which can contribute to a jetting instability. Also, the organic compounds, such as PEGs and MPD, reduce LCP viscosity in a concentration-dependent manner, and therefore, should be carefully considered when planning for an SX experiment via HVE jetting sample delivery. The analysis of the existing strategies of jet stabilization with stabilizing agents shows that Pluronic F-127 has higher stabilizing capability than monoolein and is compatible with membrane protein crystals grown using the *in meso* approach. We also present the rheometric indicators as a metric of jetting stability, which could work as an alternative or complementary experiment to the HVE jetting test.

### Practical advice on setting up rheometric experiments with LCP

We demonstrate that sample viscosity, measured with a rheometer setup, can be a reliable predictor of sample performance and optimization of HVE jetting experiments. The LCP-based samples are sensitive to ambient temperature and humidity levels, and therefore, accurate measurements should be done under controlled conditions. It is important to note several other factors: Firstly, during the sample loading, the measurement area should be fully and uniformly covered with the sample. This is especially important for the parallel plate geometry. Second, the ambient humidity around the sample plays a critical role in data reproducibility and should be kept around 80–85% to avoid sample dehydration and excess hydration. Thirdly, we recommend doing at least three consecutive measurements with the same sample to estimate the experimental errors. Fourthly, the viscosity of the ‘pure’ LCP, the monoolein: water mixture (3:2 (w/w)), is a reliable reference for LCP-based samples. To estimate the viscosity of a particular sample, monoolein should be mixed with the crystallization buffer at a 3:2 (w/w) ratio. Also, an alternative metric to the jetting stability can be used to avoid measurement setup bias. Namely, the ratio between the viscosity of the resulting medium and the empty LCP will estimate the sample stability in the HVE jetting experiment. The ratio below 0.02 corresponds to the metastable injection, and the ratio below 0.001 corresponds to the unstable injection regimes.

### Influence of ambient humidity on LCP-embedded crystals

As discussed above, ambient humidity is one of the critical parameters that should be monitored when working with LCP-based media. In the case of HVE injection, the extruded sample is not recovered after the experiment, and the time between the sample extrusion and X-ray interaction lasts less than a few seconds. Since LCP dehydration takes several minutes, it does not affect the crystal quality. The ambient humidity, however, is important for fixed-target experiments, where sample preparation and data collection can take more than 30 min. While soluble protein crystals can tolerate humidity levels lower than 80%, any LCP-embedded crystals are subject to rapid degradation caused by the lipid phase transition. The optimal humidity regime recommended for the membrane proteins is 95% relative humidity, which has been tested for multiple grown membrane proteins using *in meso* method, including the KR2 rhodopsin featured in this work.

## Methods

### Preparation of LCP mesophase samples

Monoolein (MO, > 99% purity, Nu-Check Prep) and milli-Q water (18.2 MΩ.cm, referred as water) were used to prepare the lipid cubic phase (LCP). Where specified, water was exchanged for the buffer. The additive concentration was calculated for the water fraction of the sample. The total additive concentration is also reported in Table 1. 100 μL gastight coupled syringes (Hamilton) were used for mixing the lipid with a buffer. Briefly, MO was pre-heated to 42 °C and added to one syringe, and the second syringe was filled with the buffer. The two syringes were coupled, and mixing was initiated by pushing the solvent and lipid back and forth through the coupling. The mixing process continued until the lipid mixture appeared homogeneous (at least 100 pushes). All samples were prepared on the day of the experiment unless stated otherwise.

### Rheometry experiments

**Fig. 8:**
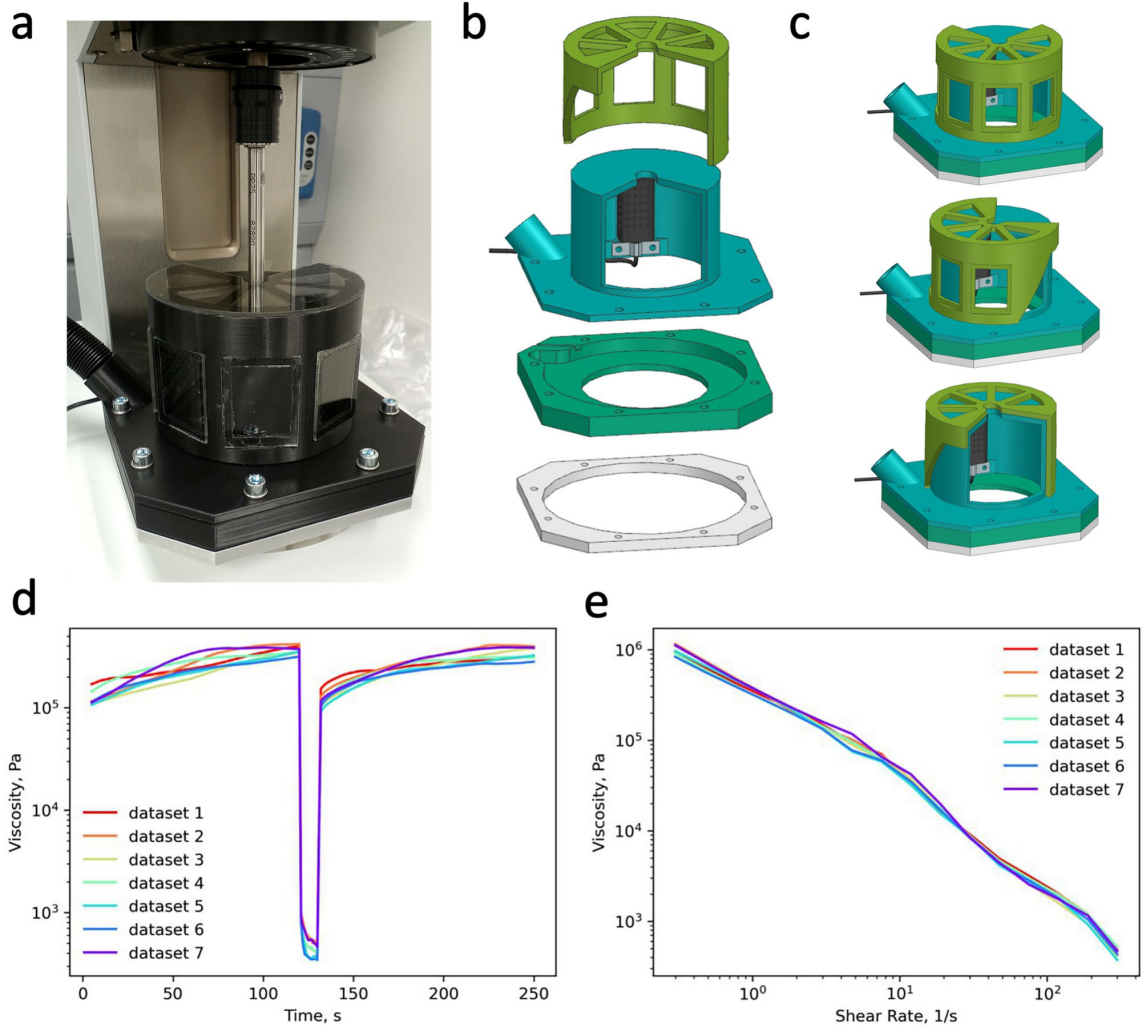
Rheometry setup used for LCP analysis. (**a**). Anton Paar rheometer MCR92 and the custom-made enclosure box connected to a humidifier system. (**b**). Schematic model of the different components of the custom-made humidity box used for rheometry measurements. (**c**). The humidity box model when it is assembled. The model was created using Siemens NX (v13.2). (**d**). Examples of the thixotropy data measured before all viscosity curves. The average humidity of the sample before and after disturbance is used to evaluate sample stability in each measurement. (**e**). Example data of multiple viscosity curves used for averaging and evaluation of sample viscosity.

Thixotropic and dynamic rheological properties were measured on a Modular Compact Rheometer (Anton Paar MCR 92) using a parallel plate geometry with a diameter of 25 mm. The distance between the plates was maintained at 300 μm. For all measurements, the temperature was maintained at 20 °C. The relative ambient humidity was maintained at 75% using a custom-made hood to prevent sample dehydration. In brief, a humidifier (SPIE SAG, KLS-205) was paired with a humidity control system (Inkbird, IHC-200) in a closed loop and connected to a custom 3D-printed box enclosed around the rheometer measuring area (see Fig. 8 for details). Two types of measurements were performed for all samples. First, the thixotropic behavior of the samples was estimated to ensure the repeatability of the experiments. For that, each sample viscosity was measured at the low shear rate (1 s^-1^) for 2 min, then the sample was disturbed at the high shear rate (300 Hz) for 10 s, followed by the recovery at the same low shear rate (1 s^-1^) for 2 min. Next, the linear viscoelastic properties of the samples were measured. The sample viscosity was measured in a shear rate (0.3–300 s^-1^) range. The thixotropic test, followed by the viscosity curve measurement, was performed multiple times (more than 3 times) to estimate the measurement errors. The exact number of measurements is provided in Source Data. The thixotropic experiment was used to remove unreliable measurement points for each measurement. The viscosity at a low shear rate before and after the disturbance was calculated and compared. The experiments with more than 20% variation between the ‘before’ and ‘after’ viscosity were excluded from the analysis. The rheometry setup calibrated for the HVE injection is available in the XBI lab of EuXFEL^28^.

### Statistics & reproducibility

Statistical parameters, including data dispersion and precision measures (mean and s.d.), are reported in Table 1. A number of experiments for each data set is provided in Source Data. Thixotropic experiment results were used to exclude data corresponding to LCP in unstabilized conditions. The number of excluded datasets and the excluded datasets are presented in Source Data. No other data was excluded from the analysis. No statistical method was used to predetermine the sample size. The experiments were not randomized. The Investigators were not blinded to allocation during experiments and outcome assessment.

### High-viscosity extrusion jet testing

The LCP injector from Arizona State University was used to extrude the sample as described in reference^46^ unless otherwise stated below. The reservoir capacity was 120 µL, with an amplification factor of 14. For sample extrusion, fused silica capillary tubing with an outer diameter of 360 µm and an inner diameter of 100 µm was used. Sample flow rates varied between 0.5 and 1.5 µL/min. The helium stream was maintained at 10 mg/min to keep the sample stream straight.

### Protein crystallization

Lysozyme crystals were obtained as described previously^37,47^. In brief, 2.5 mL of protein solution (32 mg/mL hen egg white lysozyme (Sigma) in 0.1 M sodium acetate buffer pH 3.0) and 7.5 mL precipitate solution (20% (v/v) NaCl, 6% (v/v) PEG 6000, 0.1 M sodium acetate pH 3.0) were mixed rapidly and left overnight at room temperature (24 °C on a slowly rotating wheel shaker. After gravity-induced settling, the crystalline pellet was washed several times in a crystal storage solution (0.14 M NaCl, 0.1 M sodium acetate buffer, pH 4.0). The crystals were embedded in LCP by mixing the protein crystals in their buffer with pre-melted monoolein at 42 °C at a 70:30 (v/v) ratio in 100 μL coupled gastight Hamilton syringes. The resulting LCP mesophase was homogenized before the experiment using a syringe-coupler with at least 100 slow pushes from one syringe to another. After homogenization, the crystal-containing mesophase was stored at 20 °C and remained stable without signs of phase separation.

The KR2 protein was crystallized using *in meso* approach, following the protocol previously used for BcXeR rhodopsin crystallization^48^. In particular, crystals of KR2 were grown in 200-μL PCR tubes (flat cap, ThermoFisher). For that, 2 μL of precipitant solution (3.4 M sodium malonate pH 8.0) was dispensed at the bottom of the PCR tube and covered with 12 μL of the mesophase from the gas-tight syringe. The mesophase was composed by mixing the purified KR2 protein (40 mg/mL) with the premelted at 42 °C monoolein in a 3:2 ratio (lipid: protein). The PCR tube was quickly closed and centrifuged for 1 min at 6,000 × g at 22 °C to sediment the phase to the bottom of the PCR tube. The PCR tubes were kept at 22 °C. The crystals of KR2 appeared within 2–3 weeks and reached up to 70 × 30 × 5 μm^3^ in size. The crystals from several PCR tubes were collected into a single PCR tube using centrifugation for 2 min at 20,800 × g and 22 °C. Then, the bottom part of the PCR tube with the mesophase was cut and inserted upside down into the sealed 200-μL pipette tip. The tip was sealed at the very end using a gas burner. The tip with the inserted PCR tube part was then centrifuged at 6,000 × g and 22 °C for 2 min, using a 1.5-mL tube as an adaptor for the centrifuge rotor. After centrifugation, the mesophase was found in the sealed tip. The empty PCR tube was removed, and the wide end of the tip was covered with Parafilm to avoid dehydration of the crystals. The plunger was removed from an empty, clean 100-μL gas-tight syringe, and the needle end of the syringe was sealed using Parafilm. The pipette tip with mesophase was cut from the narrow end and inserted into the back side of the prepared gas-tight syringe. The syringe with the inserted tip was fixed in the 50-mL falcon tube; for that, the syringe was wrapped in a paper towel to fit tightly into the falcon tube. Then, the tube with the syringe was centrifuged at 6,000 × g and 22 °C for 3 min to transfer the mesophase from the tip into the syringe. After centrifugation, the tip was removed, the syringe was coupled to another clean 100-μL gas-tight syringe, and the plunger was slowly inserted into the first syringe. Thus, the phase was slowly transferred into the second syringe. In the case of excessive precipitant solution and/or air, the system was temporarily uncoupled so the air or solution could be removed before homogenization of the mesophase with crystals. The mesophase was homogenized before the experiment using a syringe-coupler.

### Serial synchrotron crystallography data collection

Single-crystalline silicon chips provide a suitable scaffold material for fixed target crystallography. Chip fabrication and sample loading processes have been described in detail previously^49–51^. The HARE chips hold randomly oriented crystals in precisely defined, bottomless wells. Within a humid environment, 100–200 μL of a suspension of crystals was loaded onto a single chip by applying a vacuum suction of 0.4 mbar for approximately 30 s, as described previously^52^. The loaded chips were then covered with 2.5 μm Mylar foil to prevent evaporation, and data were collected immediately after chip loading. Serial crystallography data were collected at room temperature (24 °C) at EMBL beamline P14.2 at DESY, Hamburg, using a previously described fixed-target setup^45,46^. Chips were not rotated during an exposure; still, images were recorded at a wavelength of 0.976 Å (12.699 keV) and an exposure time of 37 ms using an EIGER X 4 M detector (Dectris, Switzerland). The sample-to-detector distance was 120 mm. The synchrotron beam had a Gaussian profile with a flux of 2 × 10^12^ photon/s and full width at half maximum (FWHM) dimensions of 20 × 7 μm^2^, with a circular collimation of 10 μm.

### Serial femtosecond crystallography data collection

Lysozyme crystals in their mother liquor were mixed with the monoolein with a ratio of 70:30 (v/v) and mixed in Hamilton syringes until the sample became homogeneous. Next, the sample was loaded into a high-viscosity injector connected to an HPLC pump^11^. The crystals were extruded into the interaction point through a 100-μm capillary at a flow rate of 3.35 μL/min. The SFX data on lysozyme crystals were collected at the SPB/SFX instrument of European XFEL during the 8376 experiment. The overview of the SPB/SFX instrument and its capabilities is presented here^53^. For this experiment, X-ray pulses with a photon energy of 12.7 keV and a pulse energy of 0.8–1.0 mJ at a repetition rate of 10 Hz were used for the experiment. The beam size was approximately 2.2 μm at the interaction point, measured on the YaG screen. The JUNGFRAU 4 M detector was used with the single memory cell (10 Hz) in the adaptive gain mode^54^.

### Structure determination and refinement

SX diffraction data were processed using CrystFEL 0.10.2^55^. Diffraction peaks were identified using the peakfinder8^56^ spot-finding algorithm with a pixel value threshold of 20 and minimum peak gradient of 50, and the signal-to-noise ratio cutoff was 5. The xgandalf algorithm was used to index reflections before three-ringed integration with radii of 2, 3, and 5 pixels. The integrated reflections were then merged and scaled with partialator using the unity model. The structures were determined by molecular replacement in Phaser^57^ using PDB IDs 3WUN and 6REX as search models for lysozyme and KR2, respectively. Structure refinement was completed using iterative cycles of Phenix.refine^58^ and manual model building in COOT^59^. Figures were prepared using PyMOL software (The PyMOL Molecular Graphics System, Version 3.1.3.1. Schrödinger, LLC) All final models contain more than 97% Ramachandran-favored amino acid residues and 0.0% outliers. Full data treatment statistics are presented in Tables 2 and 3.

## Data Availability

Atomic models built using X-ray crystallography data have been deposited in the Protein Data Bank. The KR2 structures are deposited under 9I6H and 9I6I accession codes for 95% and 85% relative humidity conditions, respectively. The lysozyme structures in its mother liquor are deposited under 9I6J, 9I6K, 9I6L, and 9I6M accession codes for 95%, 85%, 75%, and 65% relative humidity, respectively. The lysozyme structures embedded in the LCP medium are deposited under 9I6O and 9I6P accession codes for 95% and 85% relative humidity, respectively. The lysozyme structure embedded in the LCP medium via HVE injection collected at the SPB/SFX instrument of European XFEL is deposited under the 9I6N accession code. The publicly available structure of KR2 in the pentameric state (PDB ID: 6REX) was used for analysis. Data supporting the findings of this manuscript are available from the corresponding author upon reasonable request.
